# Evaluating Tumor Necrosis Factor (TNF)-Alpha Pro-inflammatory Cytokines in Healthy and Oral Squamous Cell Carcinoma Patients With and Without Diabetes

**DOI:** 10.7759/cureus.52890

**Published:** 2024-01-24

**Authors:** Ridha Azimudin, Sinduja Palati, Priyadharshini R

**Affiliations:** 1 General Pathology, Saveetha Dental College and Hospitals, Saveetha Institute of Medical and Technical Sciences, Saveetha University, Chennai, IND; 2 Oral and Maxillofacial Pathology, Saveetha Dental College and Hospitals, Saveetha Institute of Medical and Technical Sciences, Saveetha University, Chennai, IND

**Keywords:** health determinants, biomarkers, tnf- α, pro-inflammatory cytokines, oral cancers

## Abstract

Background

Tumor necrosis factor-alpha (TNF-α) has a pivotal role in the pathogenesis and prognosis of cancer as well as diabetes mellitus (DM). Many oral squamous cell carcinoma (OSCC) patients are reported to have associated comorbidities such as type 2 diabetes mellitus (T2DM). Furthermore, T2DM exaggerates inflammation due to a lack of insulin action. Therefore, OSCC patients with T2DM may progress to the advanced stage more rapidly resulting in reduced survival even after glycemic control creating a challenge to oncologists in managing these patients. Unfortunately, it is difficult to predict the course of disease in these patients just based on clinical and radiological parameters. Considering the impact of TNF alpha in both disease progression, it is an interesting biological marker to explore. Further, saliva being a noninvasive biological fluid can help measure the TNF-α levels, thereby predicating the prognosis of OSCC. Unfortunately, there is limited information about the salivary TNF-αnf levels in OSCC patients with DM.

Aim

The aim of this study was to compare the salivary TNF-α in OSCC patients with and without DM.

Methods

Saliva samples were obtained from healthy individuals, OSCC patients with DM, and OSCC patients without DM. The quantification of TNF-α levels was performed using the EliKine™ Human TNF-α ELISA Kit, an enzyme-linked immunosorbent assay. The data were reported as means and standard deviations. To assess variations in salivary TNF-α levels among these groups, the Kruskal- Wallis test was employed.

Results

The study included a total of 30 participants with 10 in each group. There were 18 males and 12 females with a mean age of 37.2± 4.7 years. The TNF-α levels between the control group (51+42±1.4 pg/ml), OSCC patients without DM (67.43 ±1.7 pg/ml), and OSCC patients with DM (268±8.5 pg/ml) were noted. The mean salivary TNF-α level was statistically higher in OSCC with DM compared to the control and OSCC without DM group.

Conclusion

The investigation compared the salivary TNF-α in OSCC patients with and without DM and has uncovered substantial differences in TNF-α concentrations within the examined cohorts, providing insights into the potential involvement of TNF-α in the context of OSCC, especially in patients with DM. Nevertheless, additional research is imperative to establish associations between TNF-α levels, the prognosis of OSCC, and the impact of DM.

## Introduction

Chronic inflammation has emerged as a significant influencer of cellular homeostasis and diverse metabolic processes, provoking genomic-level changes conducive to carcinogenesis. Oral carcinogenesis unfolds through intricate genetic events leading to sequential molecular alterations, ultimately culminating in disruptions of cellular proliferation, growth, and differentiation [1]. Inflammatory responses activate cytotoxic mediators that aid in inducing DNA damage, thereby facilitating carcinogenesis through the augmentation of genomic instability and fostering the migration and invasion of tumor cells, fueling the progression of cancer [2]. 

Crucially, pro-inflammatory cytokines, which serve as central regulators of the tumor microenvironment, are pivotal components of chronic pro-tumorigenic inflammation. These cytokines have a wide range of effects on cellular behavior, including growth, differentiation, and function. It is widely known that their physiological functions are dysregulated during inflammation and carcinogenesis. TNF-α (tumor necrosis factor-alpha) is the most important of these cytokines. TNF-α is a pleiotropic cytokine involved in inflammation, angiogenesis, programmed cell death, and proliferation, making it a key player in the malignant transformation process [3,4]. TNF-α, a multifaceted proinflammatory cytokine, is recognized for its central role in both the pathogenesis and prognosis of various diseases, including cancer and diabetes mellitus (DM) [5]. Its involvement in the modulation of immune responses, inflammation, and cell survival renders TNF-α a pivotal factor in the oncological and endocrinological domains. Importantly, the interplay of TNF-α with two seemingly distinct entities, cancer, and DM, warrants in-depth investigation.

Notably, a considerable subset of oral squamous cell carcinoma (OSCC) patients have comorbidities, with type 2 diabetes mellitus (T2DM) being a prevalent one. Prevalence of diabetes at the country level was estimated to be 7.3% and 7.7% in year 2017 and in year 2016 respectively using national population-based studies on people aged more than or equal to 20 years’ obtained from ICMR-INDIAB multi-center study [6]. This comorbidity can considerably exacerbate inflammation due to the insufficiency of insulin action, further complicating the disease course [7]. OSCC patients afflicted with T2DM may experience a more rapid progression to advanced stages, ultimately resulting in reduced survival rates, even after glycemic control has been established, thereby creating a formidable challenge for oncologists in managing these patients [8].

Traditionally, clinicians have relied on clinical and radiological parameters to predict the course of OSCC in patients. However, the disease's complexity and heterogeneity often challenge these tools' precision. This clinical challenge underscores the urgent requirement for a dependable and non-surgical biomarker, which could inform on disease progression. TNF-α is of utmost importance due to its involvement in the causation of both OSCC and diabetes of type 2. TNF-α is a main contributor to the interaction among the above-mentioned three pathologies [9]. TNF-α is a mediator that could help us understand how it impacts disease development specifically in OSCC among diabetic patients.

The choice of salivary TNF-α could lead to it being used as a possible biomarker, and this has so many benefits that it is non-invasive. Acquiring patient saliva samples is characterized by ease, lack of pain, and minimal discomfort, rendering it a viable medium for continuous longitudinal measurements. Furthermore, the absence of blood draws enhances patient compliance and reduces the likelihood of infectious exposure, thereby establishing it as a judicious approach for monitoring over an extended duration [10,11]. Routine monitoring and prognosis assessment for OSCC patients, particularly those with T2DM, are facilitated by the ease of sample collection.

While salivary TNF-α has great promise for use as of much value, there are scanty reports about the level of salivary TNF-α in diabetes-associated OSCC. The lack of data to support such an assumption further stresses the urgency to examine whether there is any relationship between the TNF-α level in the saliva and clinical outcomes in that particular patient’s group. This study, therefore, endeavors to compare the salivary TNF-α in OSCC patients with and without DM.

## Materials and methods

Study design and participants

An in vivo cross-sectional study was conducted on saliva samples from ten (n=10) healthy individuals, without systemic diseases for the control group, and for the test groups individuals diagnosed with OSCC with (n=10) and without T2DM (n=10) were recruited from the clinic. These diagnoses were established following comprehensive clinical and histopathological examinations, adhering to the World Health Organization (WHO) criteria. Informed consent was diligently obtained from all participants before proceeding with saliva collection and it was also made certain that the subject's anonymity was preserved. The study was non-invasive and simple to carry out, causing minimal discomfort to patients.

Ethical approval

The study was conducted following the approval of the Institutional Human Ethical Committee under the reference number IHEC/SDC/UG-2090/22/GPATH/233.

Criteria for selection of study subjects

The study comprised patients with and without T2DM who were clinically diagnosed and histopathologically verified to have well-differentiated OSCC. It was also made certain that no individuals with systemic comorbidities or terminally sick patients were included in the study. Patients who developed oral inflammatory disorders (e.g., dental abscess, gingivitis, periodontitis) were excluded. The investigation comprised patients with no past OSCC treatment and a history of taking drugs known to cause hyposalivation (e.g., anticholinergics, antihistamines, antihypertensives, and beta-adrenergic blockers).

For the control group, participants were selected based on their health status, absence of systemic diseases, and lack of oral lesions. Informed consent was diligently obtained from all participants before proceeding with saliva collection, and anonymity was strictly preserved. 

Patients with T2DM were included in the study, and their diabetes status was rigorously controlled. Selection criteria were based on patient history and HbA1c levels, ensuring that only individuals with controlled diabetes were included. This stringent approach aimed to minimize confounding variables related to diabetes management. TNM staging was not conducted in this study. Saliva samples were collected before the resection of the entire tumor, ensuring that the sampling process occurred consistently across all participants.

Whole unstimulated saliva (WUS) was collected from participants who were free of systemic disorders and did not have pathological abnormalities. The subjects in the study were all from the same ethnic background. All participants had filled out a questionnaire covering medical, residential, and occupational history. 

Saliva collection procedure

Sample collection for the 30 saliva samples was done from the respective individuals. Unstimulated saliva was collected from the patients in Eppendorf for a volume of 1 ml and it was stored at -20° C. Before the samples were used for the ELISA procedure, it was thawed and centrifuged.

Principle of the test

The EliKineTM Human TNF-α ELISA Kit was used to quantify TNF-α in the saliva samples collected. The competitive binding approach is used in ELISA, in which the TNF-α in the sample competes with a set amount of horseradish peroxidase (HRP)-labeled TNF-α on a human monoclonal antibody. Antibody which has been immobilized into the well binds TNF-α present in the sample, before pipetting the standards and samples into wells. Following the washing of the wells, a biotinylated anti-human TNF-α antibody was then taken. Washing off with un-bond antibiotin and adding streptavidin, conjugated with HRP in each of the respective wells. It was washed a second time, then the colored TMB substrate solution was poured following the amount of the binding TNF-α. The measurement of color intensity was done using “the stop solution,” which converted the color of the blue light from blue to yellow with a value of 450nm.

Calculation of results

The mean absorbance for each duplicate set of standards, controls, and samples was calculated, adjusting for the average zero standard optical density. Subsequently, the standard curve was generated with standard concentrations plotted on the y-axis and absorbance on the x-axis.

Statistical analysis

The statistical analysis of the study involved a comprehensive examination of the mean absorbance or Optical Density (OD) values related to TNF-α levels. The Kruskal-Wallis test, a non-parametric method suitable for comparing three or more independent groups, was employed for hypothesis testing. This analysis aimed to discern potential differences in salivary TNF-α levels among the three distinct groups of individuals under investigation. The significance level (alpha) was set a priori at 0.05, and p-values were considered statistically significant if they fell below this threshold.

The study focused on inferential statistics, particularly hypothesis testing, to evaluate whether differences in salivary TNF-α levels were statistically significant among the distinct groups of individuals under investigation. The Kruskal-Wallis test provided a robust approach for this purpose, considering the non-normal distribution of the data.

## Results

The study included a total of 30 participants. There were 18 males (60%) and 12 females (40%) and the mean age of participants in this group was 37.2± 4.7 years. The mean age in the control group was 32.8±4.8 years, that in the group OSCC without T2DM was 33.8±2.5 years, and the mean age in the group OSCC with T2DM was found to be 56.4±7.4 years. 

**Figure 1:**
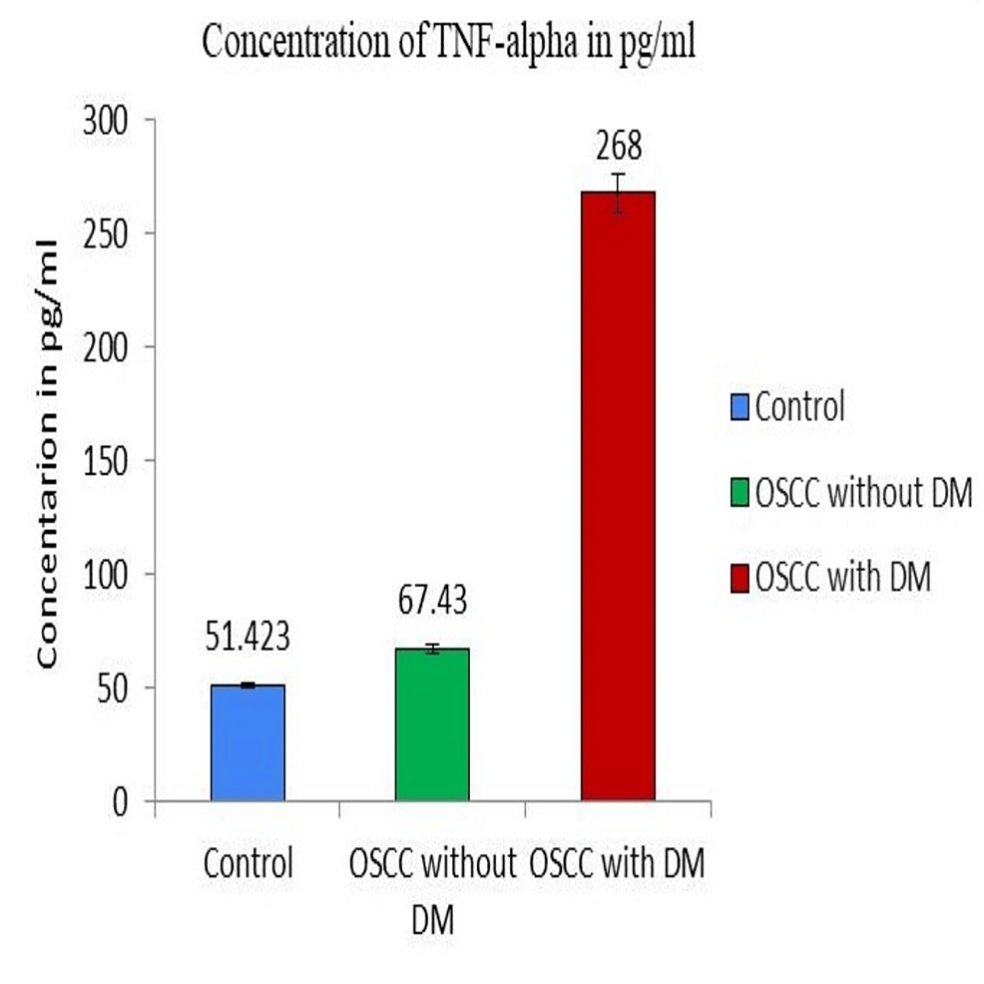
Pro-inflammatory activity of tumor necrosis factor-alpha (TNF-α) in oral squamous cell carcinoma (OSCC) patients with and without diabetes mellitus (DM) and the control group. The graph shows the five-fold increase in the concentration of salivary TNF-α in the OSCC with DM group.

The analysis revealed significant variations in the salivary TNF-α concentration across the three study groups. Specifically, there was a five-fold increase in TNF-α concentration among OSCC patients with T2DM (268±8.5 pg/ml), as illustrated in Figure 1. This finding highlights the substantial influence of pro-inflammatory cytokines, such as TNF-α, in the complex pathogenesis of OSCC.

The noticeable disparity in TNF-α levels between OSCC patients without T2DM (67.43 ±1.7 pg/ml) and the control group (51+42±1.4 pg/ml), coupled with the substantial elevation observed in OSCC patients with T2DM (268±8.5 pg/ml), supports the hypothesis that pro-inflammatory cytokines, particularly TNF-α, play pivotal roles in the pathophysiological processes underlying OSCC development and progression. The mean salivary TNF-α level was higher in OSCC with T2DM compared to the control and OSCC without T2DM group. These results underscore the importance of further investigating the mechanistic links between inflammation, T2DM, and OSCC, offering potential insights into early diagnosis and targeted therapeutic interventions.

## Discussion

Pro-inflammatory cytokines, including IL-6, IL-8, and TNF-α, serve as pivotal mediators in the intricate crosstalk between stromal and cancer cells. Their expression exhibits varying associations with tumor growth promotion or inhibition [12]. TNF-α, a proinflammatory cytokine, plays a dual role in cancer biology. On one hand, it serves as a mediator of the immune response, helping to eliminate tumor cells. 

On the other hand, it can promote carcinogenesis through its chronic and excessive production. The mechanism that facilitates the avoidance of immune surveillance is a crucial factor in oncogenesis and TNF-α mediates it. It causes an inflammatory microenvironment that supports tumor cell growth and survival [13,14]. This study aligns with existing research showing that TNF-α was highly expressed by patients with OSCC. Such an observation emphasizes the contribution that TNF-α may play in promoting oral carcinogenesis [9]. As shown by Brailo et al., patients with oral leukoplakia had significantly higher salivary levels of IL-6 and TNF-α compared to healthy controls [15]. TNF-α facilitates cell proliferation, apoptosis resistance, and the secretion of inflammatory cytokines via transcriptional activation of NF-kB. Rhodus et al. found that patients with OSCC had much higher levels of TNF α in their saliva than those with oral dysplasia [16]. It is widely noted that cytokines influence angiogenesis and inflammation.

TNF-α converting enzyme (TACE) is the enzyme responsible for proteolytic cleavage of the membrane-bound precursor protein of TNF-α to release the biologically active form of TNF-α protein (17-kDa), a process known as sheddase. In human carcinogenesis, increased expression of TACE mRNA expression has not been demonstrated to correlate with the cancer clinical stage or aggressiveness [17]. TNF-α can activate the transcriptional repressor Snail that down-regulates E-cadherin and induces the mechanism of epithelial-connective tissue transition [18]. 

Also, TNF-α linkage with various diseases such as diabetes has served to excite its link to oral carcinogenesis. Chronic inflammation can be created by increasing the production of TNF-α which is characteristic of diabetes. Chronic inflammation can also lead to the creation of pro-carcinogenic surroundings. There has been a lot of controversy regarding what links diabetes with oral cancer or how these two disorders are related. The study of the association between TNF-α, diabetes, and oral cancer may point to theragnostic targets as well as biomarkers for diagnosis [19]. Secondly, another research showed increased concentrations of TNF-α in diabetes patients suggesting the possibility of communication between disease processes [20]. Mirza et al. also observed that there is a strong correlation and elevated between TNF-α and DM [21]. The current study contributes to this data by confirming the presence of TNF-α in the saliva of OSCC patients.

The clinical implication is based on the findings on the levels of TNF-α in oral carcinogenesis and its relationship with diabetes. The presence of TNF-α within the diseased environment makes it a dependable prognostic agent for detecting oral cancer. In addition, it provides options for the exploration of therapeutics toward TNF-α.

Limitations

Although this study is a significant contribution to understanding the relationship between TNF-α in oral carcinogenesis and its relation to diabetes, several limitations should be discussed. The population studied was small and it is important to note that the results can be affected by generalizability. In other words, to strengthen the validity and generalization of the results, future studies should consider greater and more representative study groups. One important thing to note is the distinction between controlled and uncontrolled diabetes. While this study contributes to our understanding of TNF-α levels in diabetic patients, an in-depth analysis of the impact of glycemic control on TNF- expression was beyond the purview of this research. More specialized studies, which are particularly aimed at controlled and uncontrolled diabetes cohorts, will allow for a clearer understanding of the permutation within a diabetic population.

## Conclusions

The investigation compared the salivary TNF-α in OSCC patients with and without DM and has uncovered substantial differences in TNF-α concentrations within the examined cohorts, providing insights into the potential involvement of TNF-α in the context of OSCC, especially in patients with DM. 

There was a noticeable disparity in TNF-α levels between OSCC patients without T2DM and the control group, coupled with the substantial elevation observed in OSCC patients with T2DM, which supports the hypothesis that pro-inflammatory cytokines, particularly TNF-α, play pivotal roles in the pathophysiological processes underlying OSCC development and progression. To deepen the understanding of the involvement of TNF-α and other pro-inflammatory cytokines in the etiopathogenesis of OSCC, further exploration is warranted through larger-scale prospective studies and longitudinal research methods.
